# Plant-Derived Bioactive Ingredients for Osteoporosis and Bone Regeneration: Mechanisms, Pharmacology, and Delivery Strategies

**DOI:** 10.3390/cells15100935

**Published:** 2026-05-19

**Authors:** Changshun Li, Xin Zhang, Peiyu Tang, Mengying Li, Weijian Hu, Meng Zhou, Jiabin Xu

**Affiliations:** School of Stomatology, Xuzhou Medical University, Xuzhou 221004, China; lcs0626@foxmail.com (C.L.); zx19900120@foxmail.com (X.Z.); 303105120672@stu.xzhmu.edu.cn (P.T.); litianyi1993@126.com (M.L.);

**Keywords:** icariin, bone injury repair, signaling pathways, anti-inflammatory effects, drug delivery systems, angiogenesis, bioavailability

## Abstract

Icariin (ICA), a prenylated flavonoid glycoside from Epimedium (Yin Yang Huo), exhibits multi-organ pharmacological effects and has emerged as a promising candidate for osteoporosis therapy and bone tissue regeneration because of its capacity to modulate diverse osteogenic, anti-inflammatory, and angiogenic signaling pathways. Preclinical studies in osteoporotic models suggest that ICA improves trabecular microarchitecture and increases bone mineral density. Mechanistically, ICA modulates bone remodeling bidirectionally: it promotes osteoblast differentiation and extracellular matrix mineralization via activation of pro-osteogenic pathways, including Wnt/β-catenin and PI3K/Akt signaling, while simultaneously inhibiting osteoclastogenesis and bone resorption by suppressing RANKL-mediated NF-κB activation, thus reestablishing remodeling equilibrium. Despite these benefits, clinical advancement is hindered by the suboptimal oral bioavailability of ICA, stemming from poor intestinal absorption and extensive first-pass metabolism. To address this, innovative delivery systems have been engineered to enhance localized bioavailability and sustain therapeutic efficacy, such as hydrogel depots, nanoparticle formulations, and 3D-printed scaffolds enabling precise, controlled release. In bone tissue engineering applications, ICA-incorporated biomaterials—either standalone or in combination with osteogenic factors or exosomes—foster a regenerative niche by mitigating inflammation and oxidative stress, while synergistically promoting osteogenesis and angiogenesis, thereby expediting bone defect healing and osseointegration. Overall, these mechanistic elucidations and delivery advancements underscore ICA’s potential as a translational candidate for osteoporosis treatment and bone regenerative therapies. This review aims to critically and systematically synthesize current evidence on ICA-mediated bone repair and regeneration, with a particular emphasis on the molecular regulation of osteogenic signaling, the restoration of bone-remodeling homeostasis, and delivery-system-enabled strategies that may facilitate translational application.

## 1. Introduction

### 1.1. Pathological Characteristics and Clinical Challenges of Osteoporosis

Osteoporosis is a systemic metabolic bone disease characterized by low bone mass, deterioration of bone microarchitecture, and increased skeletal fragility; it is often detected only after a fragility fracture has already caused pain, limited mobility, and loss of independence [1,2]. Its high prevalence and substantial clinical burden make it a major public health concern worldwide, affecting postmenopausal women, older men, and younger individuals with primary or secondary forms of disease [3]. As populations continue to age, the absolute number of osteoporotic fractures is rising across both developed and developing regions [4]. Epidemiological data indicate that more than two million osteoporotic fractures occur annually in the United States, and that approximately one-third of women and one-fifth of men over 50 years of age worldwide will experience such fractures during their lifetime [5]. From a mechanistic perspective, osteoporosis develops through disordered bone remodeling, in which the balance between osteoblast-mediated bone formation and osteoclast-mediated bone resorption is progressively disrupted, particularly after menopause, resulting in accelerated bone loss and increased fracture susceptibility [6].

Accordingly, prevention depends largely on adequate calcium and vitamin D intake together with regular weight-bearing exercise, although calcium deficiency remains common in many populations and continues to compromise preventive effectiveness [7]. Despite considerable advances in diagnostic approaches and fracture-risk assessment, fracture prevention remains the central objective of treatment, with antiresorptive agents serving as the therapeutic mainstay and anabolic drugs providing added benefit in selected high-risk patients [8]. In current clinical practice, bisphosphonates remain the most widely used first-line agents because of their established efficacy, acceptable safety, and relatively low cost, whereas denosumab is also highly effective but requires strict adherence and careful management at treatment discontinuation [9]. Against this background, icariin (ICA), a major flavonoid glycoside isolated from Epimedium, has attracted increasing attention as a potential anti-osteoporotic agent because it promotes osteogenesis, inhibits osteoclast differentiation, and may offer multi-target therapeutic effects with relatively low production costs, thus warranting further rigorous investigation in osteoporosis management.

### 1.2. Epimedium and the Pharmacological Potential of Icariin

Epimedium, first recorded in Shennong Bencao Jing, is a traditional medicinal herb long used in compound prescriptions and complementary medicine, with reported effects on fracture healing, joint disorders, and gonadal dysfunction; more than 40 species have been identified worldwide, mainly in southwestern and central China [10]. In traditional Chinese medicine, Epimedium is regarded as an herb that dispels wind and cold, tonifies kidney yang, and strengthens tendons and bones; these functions are considered relevant to improving bone support, relieving joint pain and stiffness, and reducing wind–cold–dampness-associated Bi syndrome [11]. On this basis, Epimedium has been widely used in many regions for osteoporosis, joint disease, cardiovascular disorders, sexual dysfunction, and age-related decline, reflecting its broad medicinal value and translational potential [12].

With advances in phytochemical research, icariin has been identified as one of the major active constituents of Epimedium. Preclinical studies show that ICA exerts anti-osteoporotic, hepatoprotective, renoprotective, cardioprotective, neuroprotective, anti-inflammatory, antioxidant, and antitumor effects, highlighting its broad pharmacological potential across multiple organ systems, as summarized in Table 1 [13]. In bone metabolism, ICA promotes osteogenic differentiation, enhances mineralized matrix formation, and suppresses osteoclast-related activity, while its anti-inflammatory and antioxidant properties help reduce cytokine-mediated injury and oxidative stress [14]. Beyond bone protection, ICA has also been reported to regulate cardiovascular function, hormone balance, and immune responses, with potential relevance to multiple sclerosis, asthma, inflammatory bowel disease, rheumatoid arthritis, and cancer [15]. Compared with crude extracts or complex formulations, ICA offers clearer composition, better quality control, and greater feasibility for standardized development, supporting its potential as a standardized candidate for anti-osteoporotic drug development and clinical translation [16].

Table 1 summarizes the representative biomedical applications of icariin (ICA) across bone-related and extra-skeletal disease models, together with the corresponding study types, formulations, target systems, and principal mechanistic findings. Overall, the available evidence is predominantly derived from in vitro and preclinical in vivo studies, indicating that ICA exerts pleiotropic pharmacological activities, including anti-osteoporotic, chondroprotective, anti-inflammatory, antioxidative, neuroprotective, vasculoprotective, hepatoprotective, immunomodulatory, antiviral, and antitumor effects. Mechanistically, these effects are mainly associated with modulation of osteogenic signaling, inflammatory cascades, oxidative stress responses, mitochondrial homeostasis, ferroptosis, and survival-related pathways, such as AMPK/mTOR/autophagy, PI3K/Akt/mTOR/ULK1, TLR4–NF-κB/MAPK, JAK2–STAT3, GPER/Sirt1/HMGB1, and Nrf2–xCT/GPX4. Notably, although the table highlights the broad pharmacological spectrum of ICA, the current evidence base remains largely preclinical; therefore, these extra-skeletal activities should be interpreted primarily as a supportive systems-pharmacology context, whereas the bone-protective and bone-regenerative effects remain the central focus of this review.

### 1.3. Overview of Major Bioactive Constituents of Epimedium

**Figure 1:**
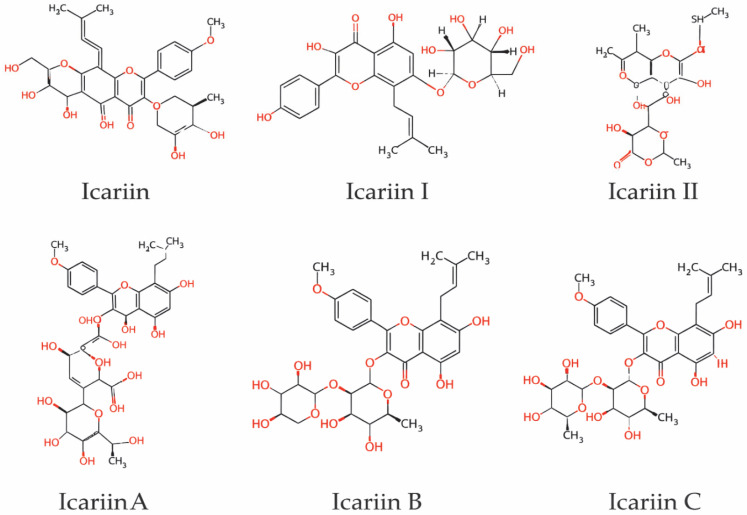
Chemical structures of icariin and its major derivatives.

Epimedium, a genus of Berberidaceae, comprises about 80 species, most of which are native to China and mainly distributed in the southwestern and central regions; *Epimedium brevicornu*, *E. sagittatum*, *E. pubescens*, and *E. koreanum* are listed in the Pharmacopoeia of the People’s Republic of China as the principal medicinal sources [41]. Phytochemical studies have identified more than 379 compounds in Epimedium, including flavonoids, lignans, organic acids, terpenoids, dihydrophenanthrene derivatives, and alkaloids, reflecting substantial chemical diversity [42]. Among these constituents, flavonoids are generally regarded as the principal bioactive constituents responsible for the pharmacological effects of Epimedium, and representative compounds include icariin A, icariin B, icariin C, icariin, baohuoside I, icariin I, and epimedin derivatives, as shown in Figure 1 [43]. In addition, Epimedium contains polysaccharides, volatile oils, and fatty acids, which together contribute to its complex chemical profile and potential multi-target pharmacological effects [44].

To date, about 60 flavonoid glycosides have been isolated from Epimedium, and more than 130 compounds have been structurally characterized, mainly including flavonoid glycosides, flavonoid aglycones such as icaritin, and other icariin-related derivatives [45]. For quality evaluation, icariin is widely used as a marker constituent because it can be consistently detected in samples from different botanical and geographic origins [46]. RP-HPLC analyses have shown marked variation in ICA content among species and samples, ranging from 0.0031% to 1.55%, with a mean of about 0.34% on a dry-weight basis, while total flavonoid content ranges from 6.43% to 12.17%, indicating considerable heterogeneity among medicinal materials [47]. Metabolic studies further suggest that flavonoid glycosides such as icariin A, B, and C can be biotransformed into icariin, one of the major metabolites, both in vitro and in vivo [48]. Mechanistically, ICA promotes osteogenesis through activation of the Wnt/β-catenin and BMP-related pathways, thereby enhancing osteoblast proliferation, differentiation, and bone formation, while also inhibiting the RANKL/NF-κB pathway to suppress osteoclast differentiation and excessive bone resorption [49]. Through this dual regulation of bone formation and resorption, ICA has become one of the most important bioactive constituents of Epimedium and a promising candidate for the treatment of osteoporosis and other bone-related disorders [50].

## 2. Chemical Properties and Pharmacokinetics of Icariin

### 2.1. Bioavailability

Although oral administration is convenient, ICA exhibits low oral bioavailability (~12%) due to poor intestinal absorption and extensive first-pass metabolism, limiting its therapeutic efficacy in bone-related disorders, as shown in Table 2 [51]. Bioavailability determines systemic exposure and directly affects therapeutic efficacy, making the improvement of ICA absorption essential for its further development in bone-related disorders [52]. Among the main determinants of oral absorption, aqueous solubility and membrane permeability are particularly important, since a drug must first dissolve in gastrointestinal fluid and then cross the epithelial barrier to reach the circulation; insufficient solubility therefore commonly leads to limited absorption and reduced pharmacological effect [53]. ICA and the related compound baohuoside II are readily soluble in several organic solvents but are nearly insoluble in water, and this poor aqueous solubility, together with weak membrane permeability, results in inadequate systemic exposure after oral administration [54].

Available studies further show that ICA has intrinsically very low aqueous solubility, approximately 0.02 mg/mL, and therefore poses a typical formulation challenge because it is both poorly soluble and poorly absorbable, as shown in Table 2 [60]. To address these limitations, recent work has mainly focused on cyclodextrin inclusion complexes, nanocarrier-based delivery systems, and modulation of crystal forms or solvates. Some systems, such as hydroxypropyl-β-cyclodextrin, can significantly increase ICA solubility under specific conditions, although the required carrier proportion is often too high for practical application [61]. Other β-cyclodextrin derivatives provide only moderate improvements, while nanocarriers appear to enhance release profiles and in vivo absorption without fundamentally overcoming the solubility barrier [62]. In addition, differences among polymorphic forms suggest that solid-state structural regulation may substantially affect dissolution performance [63].

### 2.2. Advances in Drug Delivery and Formulation

**Figure 2:**
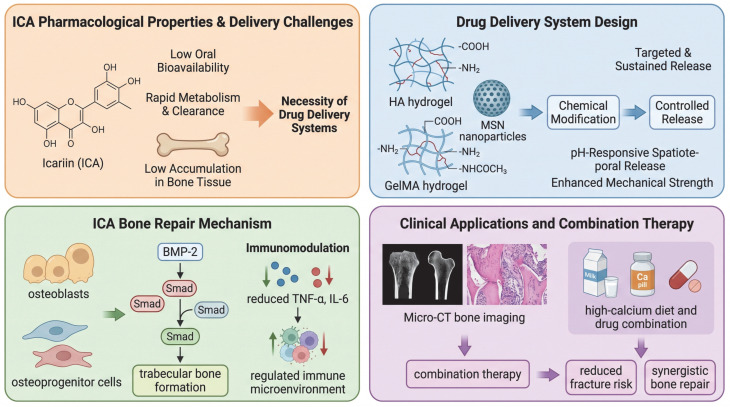
Icariin for Bone Regeneration: Challenges, Delivery Systems, and Mechanisms.

Figure 2 illustrates ICA’s pharmacokinetic challenges, including low oral bioavailability, rapid clearance, and limited bone accumulation, and highlights biomaterial-based delivery strategies—such as hydrogels, nanoparticles, and composite scaffolds—that enable sustained, targeted release and enhance osteogenic and angiogenic outcomes. Hydrogel-based carriers (e.g., hyaluronic acid and GelMA) and mesoporous silica nanoparticles (MSNs) enable chemical modification, targeted delivery, and controlled release, including pH-responsive spatiotemporal release while improving mechanical properties. Mechanistically, ICA promotes osteogenesis by activating osteoblasts and osteoprogenitor cells via BMP-2/Smad signaling, leading to enhanced trabecular bone formation. In addition, ICA modulates the immune microenvironment by reducing pro-inflammatory cytokines (e.g., TNF-α and IL-6). In preclinical studies, ICA-based therapies can be evaluated using micro-CT and histological analysis, and combination strategies (e.g., calcium supplementation) may further enhance bone repair and reduce fracture risk. In Figure 2, green arrows indicate enhanced or promoted biological effects, whereas red arrows indicate reduced or inhibited biological responses.

Icariin (ICA) has considerable potential in osteoporosis treatment and bone regeneration, but its clinical utility is restricted by low oral bioavailability, rapid metabolic clearance, and limited accumulation at target sites, resulting in insufficient local exposure and reduced efficacy [64]. Because bone repair requires prolonged drug retention and sustained local release, multiple biomaterial-based delivery systems have been developed to improve ICA stability, release kinetics and tissue retention [65]. These systems include scaffolds based on synthetic and natural polymers such as polycaprolactone, polylactic acid, poly(lactic-co-glycolic acid), silk fibroin, collagen, hyaluronic acid, and chitosan, as shown in Table 3 [66]. Among them, hyaluronic acid-based hydrogels are particularly attractive because of their good biocompatibility, biological activity, and modifiable functional groups, which support structural optimization and controlled release [67]. Through affinity modification and photocrosslinking, these hydrogels can achieve sustained drug release and in situ gel formation after injection, thereby conforming to irregular bone defects and improving their value in bone repair [68].

To improve local delivery, ICA has been incorporated into platforms such as small extracellular vesicles, gelatin microspheres, calcium phosphate bone cement, and mesoporous silica nanoparticles [88]. Composite systems based on ICA-loaded gelatin microspheres and calcium phosphate bone cement have been shown to enhance osteoinduction, promote bone formation, and reduce inflammatory responses, indicating their potential as bone substitute materials [89]. More recently, ICA-loaded mesoporous silica nanoparticles combined with gelatin methacryloyl hydrogel have been developed as injectable photocrosslinkable systems with enhanced mechanical properties and pH-responsive release [90]. These systems have been shown to promote proliferation and osteogenic differentiation of bone marrow mesenchymal stem cells in vitro and increase bone volume fraction and promote mature trabecular bone formation in vivo [91]. Overall, delivery strategies integrating biomaterial support with controlled spatiotemporal release may provide an effective approach for improving ICA retention, osteogenesis, and local bone repair, as shown in Figure 2.

### 2.3. Pharmacokinetic Characteristics of Icariin (Absorption, Distribution, Metabolism, and Excretion)

Available studies indicate that the pharmacokinetic behavior of icariin is strongly influenced by the route of administration: oral dosing shows a half-life of 3.149 ± 2.364 h, whereas intravenous administration produces much faster plasma elimination, with a half-life of 0.562 ± 0.200 h [92]. Analytical studies have further characterized its in vivo exposure: UPLC–MS/MS analysis showed no significant sex-related differences in major pharmacokinetic parameters in rats, whereas HPLC-based measurements demonstrated that after oral administration of Epimedium extract, epimedin A, epimedin B, epimedin C, and ICA reached peak plasma concentrations at about 0.25 h [93]. These findings indicate that ICA is absorbed relatively rapidly after oral administration, although its systemic exposure remains limited.

Mechanistic studies suggest that ICA is absorbed mainly in the intestine and undergoes extensive metabolic conversion, producing metabolites such as icariin I and icariin II; however, its overall oral bioavailability remains low, and its half-life in rats is reported to be about 74 min [94]. The small intestine appears to be a key site of biotransformation, where both intestinal enzymes and gut microbiota participate in its metabolism [95]. After intragastric administration, ICA and its metabolites can be detected in the stomach, small intestine, bile, urine, and feces, confirming that it undergoes absorption, distribution, metabolism, and excretion in vivo [96]. ICA remains relatively stable in the stomach, whereas higher levels of metabolites such as baohuoside II and icaritin are found in the small intestine, supporting the view that intestinal metabolism is particularly important [97]. In bile and urine, ICA together with baohuoside I and baohuoside II can be detected, while fecal samples contain multiple metabolites, mainly baohuoside II, icaritin, and demethyl-icaritin, indicating a relatively complex metabolic profile [98]. To further characterize these processes, a validated UPLC–MS/MS method has also been established for quantitative determination of ICA and multiple metabolites in mouse whole blood [99].

## 3. Mechanisms of Icariin in Promoting Osteoporosis-Related Bone Repair

### 3.1. Signaling Pathways Regulating Bone Formation and Bone Resorption

The pro-regenerative action of icariin in osteoporosis-related bone repair involves coordinated regulation of autophagy, inflammatory signaling, the immune microenvironment, and osteogenic programs [100]. In the osteoporotic and aging bone marrow niche, senescent macrophages with a senescence-associated secretory phenotype release pro-inflammatory mediators, including TNF-α, IL-1, IL-6, chemokines, and matrix metalloproteinases, thereby driving chronic low-grade inflammation and impairing the osteogenic differentiation of bone marrow stromal stem/progenitor cells, which ultimately compromises bone repair, as shown in Table 4 [101]. ICA appears to counteract this adverse microenvironment by regulating several interconnected pathways and restoring osteoimmune–osteogenic balance [101]. Mechanistically, ICA upregulates DEC1 and enhances Wnt/β-catenin signaling, while coordinated activation of the PI3K/Akt pathway further promotes osteogenic differentiation; inhibition of PI3K/Akt blocks ICA-induced DEC1 expression, supporting functional crosstalk among DEC1, Wnt/β-catenin, and PI3K/Akt signaling in bone formation, as shown in Table 4 [102].

ICA activates ERK signaling via estrogen receptors, reduces glucocorticoid-induced osteocyte apoptosis, and suppresses the RANKL-p38/ERK-NFAT axis, thereby inhibiting osteoclast differentiation and limiting bone loss, as shown in Table 4 [103]. ICA also decreases apoptosis of human bone marrow mesenchymal stem cells by inhibiting the JNK/c-Jun pathway, which further supports cell survival and osteogenic commitment, as shown in Table 4 [104]. Taken together, these findings indicate that ICA exerts anti-osteoporotic effects through dual regulation, namely promotion of osteoblast differentiation and suppression of osteoclastogenesis, particularly in ovariectomy-induced bone loss [105]. In addition, ICA may regulate bone repair through exosome-mediated intercellular communication [106]. It has been reported to promote osteoclast-derived exosome release through the MITF/RAB27A-dependent pathway and to increase miR-331-3p levels in these exosomes, which subsequently influence osteoblast activity and bone regeneration, suggesting a potential strategy for bone defect repair under complex pathological conditions, as shown in Table 4 [107].

**Table 4:** Mechanisms and signaling pathways of icariin in promoting bone regeneration and osteogenesis.

Mechanism	Signaling Pathway/Molecule	Mode of Action	Reference
Osteogenic differentiation promotion	DEC1, Wnt/β-catenin, PI3K/Akt	Promotes osteogenic differentiation and bone formation via DEC1/Wnt/β-catenin/PI3K/Akt crosstalk.	[102]
Osteocyte/BMSC survival support	Estrogen receptor (ER), ERK, JNK/c-Jun	Reduces osteocyte apoptosis and preserves BMSC survival/osteogenic commitment through ERK activation and JNK/c-Jun inhibition.	[103,104]
Osteoclastogenesis and bone resorption inhibition	RANKL-p38/ERK-NFAT axis, OPG, IL-6, TNF-α	Suppresses osteoclast differentiation and inflammatory bone resorption by inhibiting RANKL-p38/ERK-NFAT signaling, decreasing IL-6/TNF-α, and increasing OPG.	[103]
Exosome-mediated bone repair regulation	MITF/RAB27A, miR-331-3p	Enhances osteoclast-derived exosome release and osteoblast-mediated bone repair via MITF/RAB27A and exosomal miR-331-3p.	[107]
Ferroptosis inhibition and redox homeostasis	SLC7A11, GPX4, p53, ROS, MDA, GSH	Restores the SLC7A11/GPX4 axis, suppresses p53 overactivation, reduces oxidative lipid damage, and inhibits ferroptosis.	[108,109]
Matrix preservation and phenotype restoration	GPX4, MMP-3, ADAMTS-5, Collagen II, SOX9, PI3K/Akt	Preserves matrix integrity and promotes tissue repair by downregulating MMP-3/ADAMTS-5 and upregulating Collagen II/SOX9 through GPX4- and PI3K/Akt-related mechanisms.	[110]
Inflammatory injury suppression	TNF-α, ROS, NF-κB, matrix metalloproteinases	Attenuates inflammatory tissue injury by suppressing TNF-α-driven ROS, NF-κB, and MMPs.	[111,112]
Inflammation-related bone loss inhibition	COX-2, PGE2, Runx2, p38 MAPK, JNK MAPK	Protects against inflammation-associated bone loss by inhibiting COX-2/PGE2 and p38/JNK MAPK signaling while restoring Runx2.	[113]
Rheumatoid bone destruction suppression	β3 integrin, cathepsin K, MMP-9, STAT3, IL-17, Th17	Reduces rheumatoid bone destruction by suppressing osteoclast markers and the STAT3/IL-17/Th17 inflammatory axis.	[114]
Inflammasome-related response inhibition	miR-223-3p/NLRP3, TNF-α, IL-1β, IL-6	Suppresses inflammasome-associated bone damage via miR-223-3p/NLRP3 regulation and reduction in pro-inflammatory cytokines.	[115]
Pathological angiogenesis inhibition	VEGF, HIF-1α	Inhibits pathological angiogenesis in inflammatory bone-joint disorders by downregulating VEGF and HIF-1α.	[116,117,118]
Regenerative angiogenesis promotion	Autophagy, TGF-β1, VEGFA, VEGFR2, ANG1, ANG2, Tie2	Promotes vascular regeneration via autophagy-related TGF-β1 signaling and upregulation of angiogenic factors.	[119,120,121]
Ischemia-related neovascularization and angiogenic signaling	PI3K/Akt, ERK1/2, VEGF, CD31	Enhances neovascularization and blood supply reconstruction through PI3K/Akt and ERK1/2 activation with increased VEGF/CD31 expression.	[117,118]
Osteogenesis–angiogenesis coupling	VEGFA, CD31, H-type vessels	Facilitates coupled angiogenesis and bone regeneration by promoting VEGFA/CD31 expression and H-type vessel formation.	[117,119,121]

### 3.2. Anti-Inflammatory and Redox-Regulatory Mechanisms

ICA protects bone cells under inflammatory stress by restoring SLC7A11 and GPX4 expression, suppressing p53 overactivation, reducing ROS and MDA accumulation, increasing GSH levels, and alleviating mitochondrial injury, thereby inhibiting ferroptosis in osteoblasts and osteocytes, as shown in Table 4 [108]. Animal studies further show that ICA delays osteoporosis progression through activation of the SLC7A11/GPX4 axis and partial normalization of inflammatory mediators [109]. ICA also attenuates matrix degradation by downregulating MMP-3 and ADAMTS-5 while upregulating collagen II and SOX9, indicating recovery of osteogenic and chondrogenic phenotypes; these protective effects are abolished after GPX4 silencing, supporting a GPX4-dependent mechanism, as shown in Table 4 [110]. In addition, active constituents of Epimedium have been reported to enhance PI3K/Akt signaling related to matrix homeostasis, thereby promoting matrix synthesis and tissue repair [122]. Under inflammatory conditions such as osteomyelitis and chondritis, Epimedium-derived compounds can suppress TNF-α-driven ROS production, matrix metalloproteinase expression, and NF-κB activation, thereby limiting inflammatory tissue injury [111].

At the level of bone remodeling, Epimedium-related constituents inhibit lipopolysaccharide-induced bone resorption, reduce IL-6 and TNF-α expression, increase OPG expression, and antagonize RANKL signaling, thereby suppressing osteoclast-related pathways, as shown in Table 4 [123]. ICA also inhibits COX-2 and PGE2 synthesis, restores Runx2 expression, and suppresses LPS-induced activation of the p38 and JNK MAPK pathways in osteoclasts, supporting a protective effect against inflammation-related bone loss and osteoporosis, as shown in Table 4 [113]. In rheumatoid arthritis, ICA reduces inflammation and bone destruction by inhibiting osteoclast-associated markers such as β3 integrin, cathepsin K, and MMP-9, decreasing pathogenic Th17-cell responses, and suppressing STAT3-dependent IL-17 production [114]. Its protective effects are further linked to attenuation of RANKL signaling [124]. ICA also inhibits the inflammatory phenotype of rheumatoid arthritis synovial fibroblasts, lowers TNF-α, IL-1β, and IL-6 expression, and regulates the miR-223-3p/NLRP3 axis to suppress inflammasome-related responses, thereby reducing synovial fibroblast-mediated bone destruction, as shown in Table 4 [115].

### 3.3. Regulation of Angiogenesis

In osteoarthritis and other inflammatory bone-joint disorders, abnormal angiogenesis is closely associated with hypoxia and inflammatory signaling [125]. Studies have shown that vascular endothelial growth factor (VEGF) and hypoxia-inducible factor-1α (HIF-1α) are markedly elevated in osteoarthritic tissues, whereas icariin reduces both in a dose-dependent manner, indicating an inhibitory effect on pathological angiogenesis in the inflammatory joint microenvironment [116]. In contrast, under regenerative conditions, ICA exhibits pro-angiogenic activity, suggesting that its vascular effects are context-dependent [126]. In endothelial cell models, ICA promotes cell migration and tube formation, upregulates VEGFA, VEGFR2, ANG1, ANG2, and Tie2, and increases TGF-β1 expression together with autophagy activation, as shown in Table 4 [119]. Blocking autophagy weakens TGF-β1 expression and angiogenic activity, indicating that ICA promotes vascular regeneration at least partly through autophagy-related TGF-β1 signaling, as shown in Table 4 [120].

In ischemic bone disease, evidence from bone marrow microvascular endothelial cells and in vivo imaging shows that ICA enhances neovascularization and blood supply reconstruction by upregulating VEGF and CD31, activating PI3K/Akt signaling, and reducing endothelial apoptosis, thereby protecting against glucocorticoid-induced femoral head necrosis [117]. Similar effects have been observed in ischemic brain injury, where ICA, especially when combined with mesenchymal stem cells, increases VEGF expression and promotes angiogenesis through activation of the PI3K and ERK1/2 pathways [118]. In diabetic bone-defect models, ICA further increases VEGFA and CD31 expression, enhances the pro-angiogenic paracrine activity of bone marrow mesenchymal stem cells, and promotes the formation of H-type vessels at the defect site [121]. These findings indicate that ICA-mediated angiogenesis is closely linked to osteogenesis and may facilitate bone regeneration through coordinated osteogenesis-angiogenesis coupling, as shown in Table 4 [127].

## 4. Systemic Pharmacological Effects of Icariin on Major Organs

### 4.1. Effect on Liver Function

Within a systems-pharmacology framework, hepatic effects of ICA are relevant because metabolic and inflammatory regulation may indirectly influence the skeletal microenvironment [128]. Icariin has been reported to protect the liver through multiple pathways, including AKT/GSK3β, PPARα/CPT1a-ACOX1, LKB1/AMPK/ACC, and MAPK, while its anti-inflammatory activity may further contribute to hepatoprotection [129]. In metabolic dysfunction-associated fatty liver disease, particularly in models of polycystic ovary syndrome complicated by non-alcoholic fatty liver disease, ICA reduces hepatic lipid deposition by promoting fatty acid oxidation and suppressing lipogenic gene expression, thereby improving the fatty liver phenotype [130]. In high-fat diet-induced NAFLD models and free fatty acid-treated HepG2 cells, ICA lowers serum ALT, AST, TBil, TG, TC, and LDL-C levels, increases HDL-C, and reduces hepatic injury and lipid accumulation [131]. It also decreases inflammatory cytokines such as IL-1β, IL-12, and IL-6, and mechanistic studies suggest that these effects are associated with regulation of the miR-206/NF-κB/MAPK axis [132].

**Figure 3:**
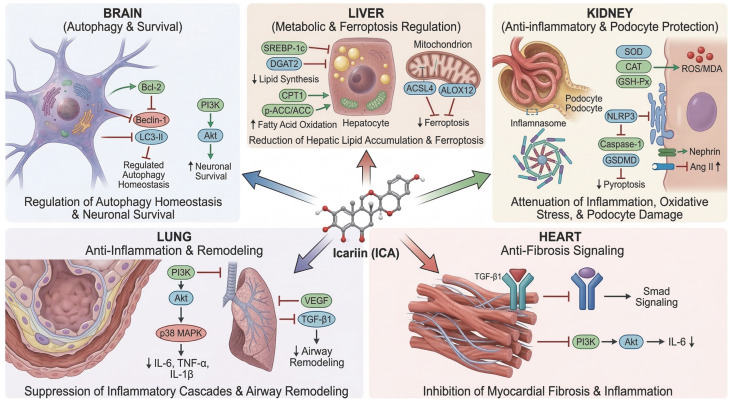
Pan-organ protective mechanisms of icariin via modulation of inflammation, metabolism, and cell-survival pathways.

Further evidence indicates that ICA improves lipid metabolic balance by downregulating lipogenic proteins such as SREBP-1c and DGAT2 and upregulating fatty acid oxidation markers including CPT1 and the p-ACC/ACC ratio, thereby attenuating hepatic steatosis, as shown in Figure 3 [133]. Beyond metabolic and inflammatory regulation, ICA also exerts hepatoprotective effects through modulation of oxidative stress and programmed cell death [134]. In a methionine-choline-deficient diet-induced non-alcoholic steatohepatitis model, ICA preserved mitochondrial structure, reduced lipid peroxidation markers such as MDA and 4-HNE, and downregulated ferroptosis-related enzymes including ACSL4 and ALOX12, suggesting that inhibition of ferroptosis may be an additional mechanism by which ICA slows the progression of liver injury, as shown in Figure 3 [135].

### 4.2. Effect on Renal Function

Icariin has shown clear renoprotective effects in multiple kidney injury models, mainly through anti-inflammatory, antioxidant, anti-apoptotic, and anti-fibrotic mechanisms [136]. In septic acute kidney injury induced by cecal ligation and puncture, ICA reduced mortality, lowered TNF-α, IL-6, and IL-1β levels in serum and renal tissue, decreased malondialdehyde accumulation, restored antioxidant enzyme activities including SOD, GSH-Px, and CAT, and alleviated renal cell apoptosis and vascular permeability, indicating substantial protection against septic renal injury, as shown in Figure 3 [137]. Further mechanistic studies using in vitro and in vivo models suggest that ICA may exert these effects through complex regulatory networks involving Acot1, Cbwd1, Ly6i, Map3k14, Mettl2, Nyap1, Set, Tmem44, and Utp20, with ceRNA analysis additionally implicating the ERK1/2, MAPK, and NF-κB signaling pathways in renal protection [138]. In unilateral ureteral obstruction models, ICA also improved oxidative stress imbalance by upregulating antioxidant proteins such as SOD1 and CAT and downregulating NOX4, while related in vitro studies showed that ICA reduced H_2_O_2_-induced injury in renal tubular epithelial cells through a SIRT1-dependent increase in catalase and peroxidase activity [139].

In models characterized by glomerular barrier injury and hemodynamic disturbance, ICA reduced systolic blood pressure and proteinuria and improved renal histopathology [140]. In L-NAME-induced renal injury, ICA reversed nephrin downregulation in podocytes, lowered circulating and renal angiotensin II levels, and alleviated mesangial expansion and glomerular damage, suggesting a protective mechanism involving podocyte preservation and suppression of the renin-angiotensin system (As shown in Figure 3) [141]. ICA also attenuated renal tubular interstitial injury by reducing the expression of NLRP3, Caspase-1, GSDMD, and IL-1β and decreasing nuclear fragmentation in tubular epithelial cells, indicating inhibition of pyroptosis-mediated inflammatory injury, as shown in Figure 3 [142]. In addition, in nephrotic syndrome-related renal remodeling, ICA reduced α-SMA expression and restored E-cadherin levels, suggesting suppression of epithelial–mesenchymal transition and fibrotic progression [143]. Collectively, these findings indicate that ICA exerts broad renoprotective effects by targeting inflammation, oxidative stress, pyroptosis, and structural remodeling. Green arrows indicate activation, upregulation, or enhancement of protective pathways, whereas red blunt-ended arrows indicate inhibition, downregulation, or suppression of pathological processes; black arrows denote downstream signaling or functional consequences, and the large colored arrows from ICA represent its organ-specific protective effects.

### 4.3. Effects on the Cardiovascular System

Cardiovascular injury is closely associated with oxidative stress, inflammation, mitochondrial dysfunction, apoptosis, and fibrosis, all of which contribute to myocardial damage and impaired cardiac function [144]. In an isoproterenol-induced heart failure model, icariin significantly attenuated hemodynamic dysfunction, myocardial apoptosis, pathological injury, ultrastructural damage, and fibrosis [145]. These protective effects were associated with inhibition of NF-κB-mediated inflammation, reduction in caspase-3-related apoptosis, enhancement of the NO-cGMP pathway, and activation of Nrf2 signaling, with overall efficacy comparable to that of sildenafil [146]. In H_2_O_2_-induced H9c2 cardiomyocyte injury models, ICA further reduced cytotoxicity and apoptosis, preserved mitochondrial membrane potential, stabilized intracellular Ca^2+^ homeostasis, and suppressed excessive ROS generation [147]. ICA also enhanced ERK/MAPK phosphorylation, and this protection was weakened by ERK inhibition, indicating that ICA improves cardiomyocyte resistance to oxidative stress through coordinated regulation of ROS clearance and ERK signaling [148].

Beyond its antioxidant and anti-apoptotic effects, ICA also limits myocardial fibrosis and improves cardiac function [149]. In dilated cardiomyopathy models, ICA inhibited TGF-β1/Smad signaling, reduced fibronectin and collagen deposition, and alleviated myocardial fibrosis, with some studies suggesting greater efficacy than metformin in improving cardiac dysfunction, as shown in Figure 3 [150]. Similar findings have been reported in myocardial infarction models, where ICA reduced TGF-β1 expression in ischemic myocardium, attenuated fibrosis, and improved cardiac function [151]. In studies related to coronary artery disease, Epimedium extract activated the PI3K/Akt pathway and reduced IL-6 expression, indicating anti-inflammatory and cardiovascular protective effects (As shown in Figure 3) [152]. In addition, ICA, as an active metabolite of Epimedium glycosides, may modulate cytokine release and promote differentiation of embryonic stem cells into cardiomyocytes, thereby contributing to myocardial repair [153].

### 4.4. Effects on the Respiratory System

In respiratory diseases, icariin has shown protective effects against both chronic and acute inflammatory lung injury by suppressing inflammatory cascades, regulating immune responses, and modulating key signaling pathways [154]. In chronic obstructive pulmonary disease (COPD) models, ICA reduced the elevated expression of IL-6, IL-1β, and TNF-α in lung tissue, improved lung function, and alleviated alveolar destruction, indicating clear anti-inflammatory and lung-protective effects (As shown in Figure 3) [155]. Mechanistic studies further showed that ICA decreased PI3K expression and reduced the phosphorylation of Akt and p38, suggesting that its effects in COPD are closely related to inhibition of the PI3K/Akt-p38 MAPK pathway (As shown in Figure 3) [156]. Similar findings were reported in another COPD study, which further confirmed that ICA ameliorates pulmonary inflammation and structural damage through suppression of the same signaling axis [157]. In asthma-related airway inflammation, ICA has also been reported to regulate macrophage polarization by decreasing M1 macrophages and increasing M2 macrophages, thereby alleviating airway inflammation; potential targets may include Jun, Jak2, Syk, Tnf, and nitric oxide synthase-related pathways [158].

Beyond inflammatory control, ICA also shows beneficial effects on airway remodeling and acute lung injury [159]. In asthmatic models, ICA improved lung function, reduced airway inflammation and remodeling, and downregulated VEGF and TGF-β1 expression, suggesting inhibition of remodeling-associated proliferative signaling (As shown in Figure 3) [160]. In lipopolysaccharide-induced acute lung injury, ICA reduced neutrophil and macrophage infiltration, lowered inflammatory cytokines, chemokines, and adhesion molecules, and significantly improved lung tissue injury and functional impairment [161]. In bilateral adrenalectomy-associated acute lung injury, ICA showed anti-inflammatory and lung-protective effects comparable to dexamethasone, but without suppressing endogenous corticosterone production or inhibiting the hypothalamic–pituitary–adrenal axis [162]. This finding suggests that ICA may possess a more favorable endocrine safety profile while retaining therapeutic potential in acute inflammatory lung injury [163].

### 4.5. Effects on the Central Nervous System

Icariin has shown neuroprotective effects in multiple models of central nervous system injury and neurodegeneration, acting through regulation of synaptic plasticity, autophagy, oxidative stress, inflammation, and pro-survival signaling [164]. In traumatic brain injury models, ICA increased brain-derived neurotrophic factor, synaptophysin, and postsynaptic density protein 95 expression, improved sensory-motor and cognitive function, and thereby promoted functional recovery mainly through enhancement of neuroplasticity rather than direct reduction in neuronal loss [165]. Further studies showed that ICA restored hippocampal histone H3 acetylation, increased acetylcholine content and choline acetyltransferase activity, and improved post-traumatic cognitive impairment, suggesting additional involvement of epigenetic regulation and cholinergic function [166]. In neurodegenerative and aging-related models, ICA alleviated Alzheimer-like cognitive deficits, reduced excessive autophagy by regulating Bcl-2, Beclin-1, and LC3-II, and improved brain autophagic homeostasis through the AMPK/mTOR/ULK1 pathway (As shown in Figure 3) [167].

In ischemic brain injury, ICA reduced lactate dehydrogenase release and malondialdehyde levels, increased superoxide dismutase activity, enhanced endothelial nitric oxide synthase expression, and attenuated oxidative DNA damage, thereby reducing brain edema and alleviating injury after middle cerebral artery occlusion; these effects may be related to activation of SIRT1-dependent PGC-1α signaling [168]. In ischemia–reperfusion models, ICA further reduced neurological deficit scores and infarct volume, decreased IL-1β and TGF-β1 expression, inhibited IκB-α degradation and NF-κB activation, and increased PPARα and PPARγ expression, indicating combined anti-inflammatory and endogenous protective effects [169]. In neonatal hypoxic–ischemic brain injury, ICA showed antioxidant, anti-inflammatory, and anti-apoptotic actions in vivo and in vitro, promoted PI3K/Akt-mediated survival signaling, inhibited pro-apoptotic pathways, and improved neurobehavioral outcomes, suggesting potential value in perinatal brain injury, as shown in Figure 3 [170].

This schematic illustrates the proposed protective actions of icariin in the brain, liver, kidney, lung, and heart. In the brain, ICA may promote neuronal survival by maintaining autophagy homeostasis through regulation of the Bcl-2/Beclin-1/LC3-II axis and activation of the PI3K/Akt signaling pathway. In the liver, ICA appears to alleviate hepatic lipid accumulation and ferroptotic injury by downregulating lipogenic mediators, including SREBP-1c and DGAT2, while enhancing fatty acid oxidation via CPT1 and p-ACC/ACC signaling. ICA may also suppress ferroptosis-related regulators such as ACSL4 and ALOX12, thereby preserving mitochondrial integrity and metabolic homeostasis. In the kidney, ICA exerts renoprotective effects by attenuating oxidative stress, inflammation, and podocyte injury. These effects are associated with increased antioxidant capacity, as reflected by SOD, CAT, and GSH-Px activity, reduced ROS/MDA accumulation, inhibition of NLRP3 inflammasome activation and caspase-1/GSDMD-mediated pyroptosis, preservation of nephrin expression, and mitigation of angiotensin II-associated injury. In the lung, ICA suppresses inflammatory responses and airway remodeling by modulating the PI3K/Akt/p38 MAPK pathway, decreasing the expression of pro-inflammatory cytokines, including IL-6, TNF-α, and IL-1β, and inhibiting VEGF- and TGF-β1-driven remodeling processes. In the heart, ICA may attenuate myocardial fibrosis and inflammation primarily by inhibiting TGF-β1/Smad signaling and regulating the PI3K/Akt pathway, accompanied by reduced IL-6 expression. Collectively, ICA exhibits broad cytoprotective potential through coordinated regulation of autophagy, lipid metabolism, oxidative stress, inflammasome activation, ferroptosis, pyroptosis, inflammatory signaling, and fibrotic remodeling.

## 5. Efficacy and Safety Evaluation of Icariin in Osteoporotic Animal Models

### 5.1. Animal Studies on the Effectiveness of Icariin in Improving Osteoporosis

**Figure 4:**
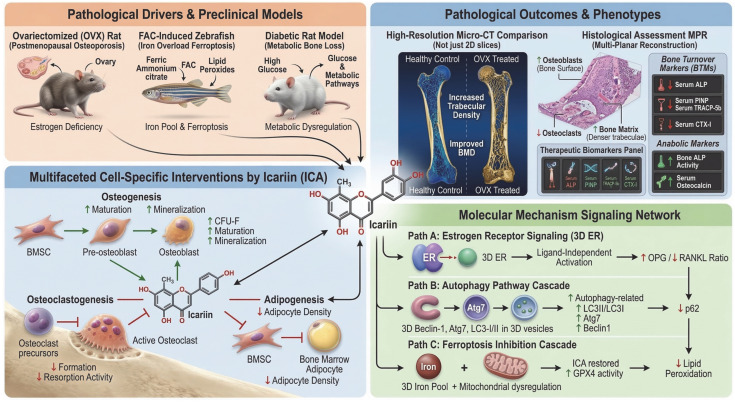
Mechanisms Underlying the Anti-osteoporotic Effects of Icariin in Animal Models: Multi-pathway Regulation.

The ovariectomized (OVX) rat is the most widely used model of postmenopausal osteoporosis, and icariin, the principal flavonoid glycoside of Epimedium, has shown clear anti-osteoporotic effects in this setting, as shown in Figure 4. Previous studies indicate that Epimedium inhibits bone resorption and promotes bone formation, thereby exerting an overall bone-protective effect in experimental osteoporosis [171]. In OVX rats, oral Epimedium extract increased trabecular formation in tibial cancellous bone, reduced trabecular separation, and lowered urinary calcium excretion and serum alkaline phosphatase levels, suggesting suppression of bone resorption; these effects were considered to be associated with estrogen-related signaling, as shown in Figure 4 [172]. Further studies showed that ICA reduced bone loss and loss of bone strength in the distal femur and increased the OPG/RANKL mRNA ratio in tibial tissue, indicating inhibition of osteoclast-related bone resorption [173]. Notably, ICA prevented OVX-induced bone loss without inducing uterine hyperplasia, suggesting a safer estrogen receptor-related bone-protective profile [174]. ICA also improved the high-turnover state induced by OVX by reducing serum ALP, PINP, TRACP-5b, and CTX-I levels, while increasing the LC3-II/LC3-I ratio and the expression of Atg7 and Beclin-1 and decreasing p62 expression in femoral tissue, indicating a role in restoring autophagy-related bone phenotypes, as shown in Figure 4 [175].

At the cellular level, ICA enhanced osteogenic differentiation of rat bone marrow mesenchymal stem cells, promoted osteoblast maturation and mineralization, and inhibited osteoclastogenesis and bone resorption in vivo [176]. In vitro, ICA increased alkaline phosphatase activity, osteocalcin secretion, calcium deposition, and the number of alkaline phosphatase-positive fibroblast colonies, supporting its anabolic effect on bone formation [177]. Its bone-protective activity has also been confirmed in non-rodent models; for example, in Japanese medaka, ICA alleviated RANKL-induced bone injury and showed efficacy comparable to alendronate in protecting mineralization, as shown in Figure 4 [178]. In iron overload-related osteoporosis, evidence from ferric ammonium citrate-induced zebrafish models suggests that ICA may exert anti-osteoporotic effects partly by inhibiting lipid peroxidation and ferroptosis-related damage [179]. In addition, in diabetic rats, prolonged ICA administration improved bone mineral density, increased trabecular thickness and bone volume fraction, reduced osteoclast numbers, and decreased bone marrow adipocyte density, indicating a protective effect against diabetes-related bone loss, as shown in Figure 4 [180].

This schematic summarizes the pathological drivers, cellular targets, phenotypic outcomes, and molecular mechanisms underlying the osteoprotective effects of icariin. Ovariectomized rats, ferric ammonium citrate-induced zebrafish, and diabetic rats represent bone-loss models associated with estrogen deficiency, iron overload-related ferroptosis, and metabolic dysregulation, respectively. ICA promotes bone formation by enhancing the osteogenic differentiation of bone marrow stromal cells, increasing osteoblast maturation, colony-forming capacity, and matrix mineralization. In parallel, ICA suppresses osteoclastogenesis and bone-resorptive activity, while limiting adipogenic differentiation and bone marrow adipocyte accumulation.

At the tissue level, ICA improves bone microarchitecture, as evidenced by increased trabecular density, enhanced bone mineral density, greater osteoblast abundance, reduced osteoclast activity, and denser trabecular matrix formation. These effects are accompanied by favorable regulation of bone turnover biomarkers, including decreased catabolic markers such as TRACP-5b and CTX-I, and increased anabolic markers such as bone-specific alkaline phosphatase and osteocalcin. Mechanistically, ICA appears to coordinate estrogen receptor signaling, autophagy, and ferroptosis inhibition. ICA-mediated estrogen receptor activation increases the OPG/RANKL ratio, thereby restraining osteoclast differentiation. ICA also enhances autophagic activity, characterized by upregulation of Beclin-1, Atg7, and LC3-II/LC3-I and downregulation of p62. In addition, ICA alleviates iron overload- and mitochondrial dysfunction-associated ferroptosis by restoring GPX4 activity and reducing lipid peroxidation. Collectively, ICA preserves skeletal integrity by promoting osteogenesis, inhibiting osteoclastogenesis, reducing marrow adipogenesis, and attenuating ferroptotic injury under pathological bone-loss conditions. In the Figure 4, red downward arrows denote decreases or inhibition of the indicated processes, while green upward arrows denote increases or enhancement of the indicated biological activities.

### 5.2. Effective Dose Range of Icariin for Anti-Osteoporotic Therapy

Preclinical studies demonstrate a dose-dependent anti-osteoporotic effect of ICA, indicating the presence of an optimal therapeutic window; variability in outcomes is influenced by administration route, treatment duration, disease severity, and pharmacokinetics [181]. In the classical ovariectomized (OVX) model, dietary ICA given for 3 months at 50, 500, and 3000 ppm significantly improved bone mass and microarchitecture, with the intermediate dose of 500 ppm showing the most consistent overall benefit, whereas the highest dose did not produce a proportional increase in efficacy, suggesting the presence of an optimal therapeutic window rather than a simple linear dose–response relationship [182]. A related 12-week pair-fed study using the same dose gradients further showed that all three doses improved bone mineral density and other skeletal indices, corresponding approximately to 3.26, 32.61, and 195.65 mg/kg/day, respectively, and providing a practical reference for dose comparison across studies [183]. In addition, ICA at 25 mg/kg/day effectively prevented bone loss in OVX rats while preserving uterine weight and histological features comparable to sham controls, suggesting a favorable balance between bone efficacy and estrogen-related safety at moderate doses [184].

In more severe models, such as OVX combined with glucocorticoid-induced bone loss, the influence of formulation design and administration route becomes more evident [185]. At the same ICA dose of 90 mg/kg, delivery system-encapsulated ICA produced greater improvements than free ICA in bone formation, bone resorption, microarchitecture, mechanical performance, and histological outcomes, indicating that optimized delivery can enhance pharmacological efficacy by improving local exposure and tissue distribution [186]. Interpretation of the effective dose range may also be affected by disease-related changes in metabolism. In osteoporotic rats, reduced hydrolysis of ICA and related compounds by gut microbiota and duodenal enzymes suggests impaired biotransformation and absorption under pathological conditions, implying that the same oral dose may yield different systemic exposure in different disease states [187]. In aging-related intervention studies, continuous intragastric ICA administration for 60 days in aged mice fed a high-calcium diet increased femoral bending load and elastic modulus, indicating improvement not only in bone mass but also in bone quality and mechanical strength [188]. These findings suggest that ICA, particularly when combined with adequate calcium intake, may help reduce fracture risk by improving both trabecular density and cortical bone strength.

### 5.3. Challenges in Drug Delivery: Design and Implementation Strategies

Although icariin has considerable potential in bone metabolic disorders, its diglycoside structure is associated with low bioavailability, which limits tissue exposure and makes it difficult to achieve effective concentrations in bone [189]. This pharmacokinetic limitation has driven the development of new delivery systems to improve ICA stability, loading efficiency, local retention, and therapeutic efficacy in osteoporosis and related conditions [190]. Recent studies have established a range of inorganic and organic delivery platforms for this purpose. Among them, hydroxyapatite (HAp)@ICA composites have been regarded as an important advance because they improve ICA stability and bioavailability while supporting osteoblast activity and bone microarchitecture [191]. ICA has also been incorporated into porous coordination framework nanoparticles to generate ICA@PCOF systems, which exhibited favorable physicochemical properties and dual functions in vitro, namely regulation of macrophage polarization and promotion of osteogenic differentiation, supporting their value as immunomodulatory and osteogenic delivery platforms [192]. In addition, strontium-modified bioactive glass submicron spheres have been used as co-delivery systems for strontium ions and ICA, with higher strontium content associated with greater ICA loading and faster release, while also enhancing the osteogenic differentiation of bone marrow mesenchymal stem cells from osteoporotic rats [193].

In bone tissue engineering, biomimetic carriers have further expanded the application of ICA [194]. BioCaP-based systems loaded with ICA, particularly in combination with BMP-2, enhanced osteogenic differentiation and mineralization in vitro and promoted new bone formation in rat calvarial defect models, indicating that ICA can strengthen the osteogenic performance of mineralized scaffolds and act synergistically with classical growth factors [195]. From the perspective of osteogenesis-angiogenesis coupling, ICA has also been loaded into rod-shaped micro/nano-hydroxyapatite carriers, where it enhanced osteogenic marker expression, increased VEGF and ANG1 levels, activated AKT signaling, and promoted both new bone formation and angiogenesis in femoral defect models [196]. In addition, ICA-chitosan/hydroxyapatite composite systems have been developed to achieve sustained and controllable release, while preserving ICA bioactivity, increasing alkaline phosphatase activity, and inducing mineralized nodule formation in stromal cells [197]. Taken together, these studies indicate that rational delivery design is essential for overcoming the bioavailability limitations of ICA and for translating its osteogenic and bone-reparative effects into effective therapeutic strategies [198].

### 5.4. Monitoring of Therapeutic Efficacy and Safety

Icariin exerts anti-osteoporotic effects through multiple mechanisms, including suppression of inflammation, promotion of angiogenesis, improvement of tissue repair under metabolic stress, and regulation of bone marrow mesenchymal stem cell fate [199]. Experimental Studies have shown that ICA downregulates peroxisome proliferator-activated receptor γ (PPARγ), inhibits adipogenic differentiation of BMSCs, preserves osteogenic potential, and promotes endothelial cell migration and proliferation, thereby supporting bone regeneration through both osteogenesis and neovascularization [200]. Clinical evidence from systematic reviews and meta-analyses indicates that, compared with conventional Western medicine alone, regimens combining icariin-related traditional Chinese medicine with standard therapy significantly improve lumbar spine and femoral neck bone mineral density and reduce pain scores [201]. These benefits are accompanied by reduced CTX-1 and increased osteocalcin, whereas no consistent improvement has been shown for PINP or fracture incidence [202]. Safety analyses generally show no significant increase in overall adverse events, although some studies suggest that specific formulations or prolonged treatment may increase risk, underscoring the need for standardized safety reporting and longer-term follow-up [203].

Similar meta-analytic evidence has confirmed improved bone mineral density and pain relief with combined therapy but no clear reduction in fracture incidence, emphasizing the need for unified endpoints, standardized dosing strategies, and long-term monitoring in future studies [204]. Preclinical findings are consistent with these observations. A systematic review of ovariectomized rat models showed that ICA significantly increased femoral and lumbar bone mineral density and improved trabecular histomorphometric indices, including trabecular area, thickness, and separation [205]. In addition, oral ICA in ovariectomized rats improved bone loss by regulating osteogenesis-related markers and the RANKL/OPG ratio, while dose-dependently inhibiting osteoclast formation and bone resorption and promoting osteoclast apoptosis [206]. Research on combined interventions using Epimedium with stem cells or exercise remains limited, although other flavonoids such as naringenin, quercetin, rutin derivatives, and anthocyanins have also shown bone-protective effects through related mechanisms, including inhibition of MAPK signaling, suppression of osteoclast activity, and promotion of osteoblastogenesis [207].

## 6. Future Perspectives

Future research is likely to focus on biohydrogel-based icariin delivery systems to improve therapeutic efficacy in osteoporosis [208]. In bone tissue engineering, ICA-loaded carriers with biomimetic bone-like architecture are expected to receive increasing attention, although optimization of mechanical strength and sustained-release performance remains necessary [209]. Advanced manufacturing methods, especially 3D printing, further expand scaffold design possibilities. For example, porous scaffolds incorporating strontium-doped mesoporous bioactive glass and polycaprolactone have enabled co-release of Sr^2+^ and ICA, thereby promoting osteogenic differentiation and inhibiting osteoclast activity [210]. Likewise, multilayer titanium alloy scaffolds with controlled release of ICA and Mg^2+^ have been shown to induce macrophage polarization toward the reparative M2 phenotype and improve osseointegration [211]. These biomimetic scaffold strategies may therefore provide a practical approach not only for restoring bone mass but also for managing osteoporotic bone defects.

A central obstacle to clinical translation remains the unfavorable pharmacokinetic profile of ICA, including low oral bioavailability and short plasma half-life, which make it difficult to maintain effective concentrations in bone tissue [212]. For this reason, targeted delivery and sustained-release technologies are likely to become increasingly important [213]. Biomaterial-based platforms such as smart hydrogels, nanocarriers, and 3D-printed scaffolds can improve local accumulation of ICA, prolong drug release, and enhance targeting efficiency [214]. By controlling spatiotemporal release within the bone microenvironment, these systems may better coordinate suppression of inflammation with promotion of osteogenesis, thereby improving therapeutic performance [215]. For example, photocrosslinked hyaluronic acid-based hydrogels containing ICA derivatives have achieved prolonged release while preserving biological activity, providing a promising strategy for formulation optimization [216]. Injectable hydrogels are particularly attractive because they allow minimally invasive delivery, in situ gelation, filling of irregular bone defects, and sustained local drug release [217]. Materials such as gelatin methacrylate, gelatin, chitosan, and hyaluronic acid offer favorable biocompatibility and biodegradability, while also serving as structural supports for new bone formation [218]. ICA-loaded mesoporous silica nanoparticle/GelMA composite hydrogels have shown improved compressive strength, pH-responsive release, enhanced proliferation and osteogenic differentiation of bone marrow mesenchymal stem cells, and greater new bone formation in vivo [219].

Stimuli-responsive systems may further improve ICA delivery by adapting release to the pathological bone microenvironment [220]. Because osteoporotic lesions are often associated with acidification and oxidative stress, hydrogels that respond to pH, temperature, light, or other cues can provide more precise, demand-responsive release [221]. For example, MSN-GelMA composite hydrogels accelerate ICA release under acidic conditions, while near-infrared-responsive systems have been designed to co-release ICA and selenium, thereby simultaneously reducing reactive oxygen species and inflammatory factors [222]. Thermosensitive hydrogels permit injection in liquid form followed by in situ solidification at body temperature, whereas photosensitive systems allow external regulation of release timing and dose [223]. Through such designs, ICA carriers may achieve better spatiotemporal control and improved treatment safety [224]. In parallel, combined delivery strategies are emerging as another key direction. Co-delivery of ICA with BMP-2 has shown clear synergistic effects in bone regeneration, increasing new bone formation more effectively than single-agent systems in critical-sized defect models [225,226]. In addition, exosome-based strategies are attracting interest because exosomes provide biological signals that promote osteogenesis and angiogenesis. Plant-derived or cell-derived exosomes may therefore complement ICA pharmacology and enhance the local repair microenvironment [227]. Future studies may also explore co-delivery of ICA with osteogenesis-related miRNAs, as previous work suggests that ICA can regulate stem cell osteogenic differentiation through miRNA-related pathways [228].

Overall, integration of ICA into biohydrogel and scaffold-based delivery systems is shifting osteoporosis treatment from simple improvement of bone mineral density toward active bone regeneration. This trend highlights the growing convergence of biomaterials science, pharmacology, and bone biology [229]. However, several issues remain unresolved, including carrier biocompatibility, metabolic safety, scaffold mechanics, and the balance between structural support and release kinetics [230]. In addition, rigorously designed animal studies and large-scale clinical trials are still required to define the long-term efficacy and safety of ICA-loaded delivery systems [231]. With continued progress, these approaches may help overcome the current barriers to ICA translation and support its development into a new generation of biomaterial-based therapeutics for osteoporosis [232].

## Figures and Tables

**Table 1:** Biomedical applications of ICA across bone and extra-skeletal models.

Biomedical Application	Study Type	Target Organ/Disease Model	Formulation Used	Main Findings	Reference
Bone protection/anti-osteoporosis	In vivo and in vitro	OVX mice; BMSCs (bone marrow mesenchymal stem cells)	ICA monomer	Promoted osteogenesis and reduced marrow adiposity via AMPK–mTOR–autophagy.	[17,18]
Cartilage homeostasis/osteoarthritis	In vitro	IL-1β-induced SW1353 chondrocytes	ICA monomer pretreatment	Preserved cartilage matrix homeostasis via PI3K/Akt/mTOR/ULK1.	[19,20]
Anti-inflammatory	In vivo	LPS-induced acute lung injury	ICA monomer in 0.5% CMC-Na	Suppressed C5a–C5aR1, TLR4–NF-κB/MAPK, and JAK2–STAT3 signaling.	[21,22]
Antioxidative	In vitro	Glutamate-injured SH-SY5Y cells	ICA monomer	Reduced oxidative stress and mitochondrial injury by inhibiting JNK/p38-related apoptosis.	[23,24]
Neuroprotection	In vivo and in vitro	AD-like rats and NSCs	ICA monomer; oral ICA combined with NSC transplantation	Enhanced NSC survival, proliferation, migration, and neuronal differentiation.	[25]
Antidepressant-like effect	In vivo	Prenatal stress offspring rats	ICA monomer	Improved depressive-like behavior, synaptic plasticity, and mitochondrial homeostasis.	[26,27]
Improvement of cardiovascular function/vasculoprotection	In vivo and in vitro	Diabetic rat aortas; high glucose-induced HUVECs	ICA monomer	Improved endothelial function via GPER/Sirt1/HMGB1.	[28,29]
Myocardial ischemia/reperfusion injury	In vitro	OGD/R-induced H9C2 cells	ICA monomer	Protected cardiomyocytes by inhibiting IRE1/JNK-mediated ER stress and ferroptosis coupling.	[30]
Atherosclerosis	In vivo and in vitro	ApoE mice; ox-LDL-stimulated VSMCs	ICA monomer in CMC-Na	Attenuated plaque formation and suppressed VSMC proliferation/migration.	[31,32]
Hormonal regulation/endocrine modulation	In vivo and in vitro	Leydig cells; DEHP-exposed male rats	ICA monomer	Promoted steroidogenesis via Esr1/Src/Akt/Creb/Sf-1.	[33]
Immune response enhancement/immunopotentiation	In vivo and in vitro	Colorectal cancer cells; MC38 tumor-bearing mice	ICA combined with anti-PD-1 therapy	Enhanced anti-PD-1 efficacy by inducing ferroptosis and strengthening CD8+ T-cell immunity.	[34,35]
Hepatoprotection/anti- hepatic injury	In vivo	MCD diet-induced NASH mice	ICA monomer in 0.5% CMC-Na	Alleviated liver injury by suppressing ferroptosis via Nrf2–xCT/GPX4.	[36,37]
Antiviral	In vitro	DHAV-1-infected duck embryonic hepatocytes	ICA/pICA	Reduced oxidative stress, inflammation, and apoptosis; pICA showed stronger activity.	[38]
Antitumor	In vivo and in vitro	TNBC cells; 4T1 tumor-bearing mice	ICA monomer	Inhibited tumor growth and induced autophagy/apoptosis via AMPK/mTOR/ULK1.	[39,40]

**Table 2:** Solubility, bioavailability, and molecular weight of ICA and its derivatives.

Compound	Representative Solubility	Bioavailability	Molecular Weight (g/mol)	Reference
Icariin	Poor aqueous solubility (~0.02 mg/mL)	Low oral bioavailability (~12%)	676.66–676.70	[51]
Icariin I	Low aqueous solubility; DMSO-soluble	Low oral bioavailability; limited absolute bioavailability data	530.52	[55]
Icariin II	Very low water solubility (~49.6 μg/mL)	Low oral bioavailability (~2%)	514.52	[56]
Icariin A	Low aqueous solubility; usually DMSO-formulated	Bioavailability data are scarce; overall exposure is low	836.8–838.8	[57]
Icariin B	Low aqueous solubility	Very poor oral bioavailability	808.8	[58]
Icariin C	Low-to-moderate aqueous solubility (~1 mg/mL in PBS)	Very low oral bioavailability (~0.58%)	822.80	[59]

**Table 3:** Key properties and limitations of biomaterials for bone tissue engineering.

Material	Biocompatibility	Degradability	Mechanical Properties	Main Advantages	Main Limitations	Reference
Polycaprolactone (PCL)	Good; mild inflammation	Slow; long-term support	Tough, ductile; moderate stiffness	Excellent processability; printable/electrospinnable	Hydrophobic; poor cell affinity; overly slow degradation	[69]
Polylactic acid (PLA)	Good; clinically used	Moderate–slow; acidic byproducts	High strength/stiffness; brittle	Good structural support; easy fabrication	Brittleness; local acidification	[70,71]
Poly (lactic-co-glycolic acid) (PLGA)	Excellent; well established	Tunable; faster than PLA/PCL	Moderate; declines during degradation	Adjustable degradation; drug delivery/scaffolds	Acidic degradation; limited long-term stability	[72,73]
Silk fibroin (SF)	Excellent; cell-supportive	Enzymatic; structure-dependent	Strong and tough	Bioactive; versatile processing	Batch variability; complex degradation control	[74]
Collagen	Excellent; low immunogenicity	Rapid enzymatic degradation	Weak; poor stability	High bioactivity; cell-instructive	Weak mechanical properties; fast degradation	[75,76]
Hyaluronic acid (HA)	Excellent; highly hydrophilic	Rapid enzymatic degradation	Weak; non-load-bearing	ECM-mimetic; lubricating; hydrating	Poor mechanical stability; short residence	[77,78]
Chitosan (CS)	Good; antibacterial; bioadhesive	Biodegradable; MW/DD-dependent	Moderate–weak; brittle	Hemostatic; antibacterial; modifiable	pH-dependent solubility; low strength	[79,80]
Hyaluronic acid-based hydrogel	Good; ECM-like	Tunable by crosslinking	Soft, viscoelastic	Injectable; cell delivery; soft tissue repair	Low strength; limited durability	[81]
Gelatin microspheres	Good cytocompatibility	Fast; crosslinking-tunable	Limited particle stability	Controlled release; easy fabrication	Swelling/collapse; poor support	[82,83]
Calcium phosphate cement (CPC)	Excellent; osteoconductive	Slow resorption	Good compression; brittle	Injectable; moldable; bone defect filling	Poor tensile/flexural properties	[84]
Mesoporous silica nanoparticles (MSNs)	Generally good; context-dependent	Slow silicate degradation	Non-load-bearing	High loading; easy functionalization	Dose/surface-dependent toxicity risk	[85]
Gelatin methacryloyl hydrogel (GelMA)	Good; cell-adhesive motifs retained	Tunable enzymatic degradation	Improved mechanical strength but still relatively soft	Photocrosslinkable; bioprintable	Limited strength; photoinitiator concerns	[86,87]

## Data Availability

No new data were created or analyzed in this study. Data sharing is not applicable to this article.
